# Validation of a core beliefs model of disordered eating in adults with an eating disorder

**DOI:** 10.1007/s40519-025-01787-4

**Published:** 2025-10-02

**Authors:** Amaani H. Hatoum, Amy L. Burton, Maree J. Abbott

**Affiliations:** 1 School of Psychology, University of Sydney, Level 2, 94 Mallet Street, Camperdown, NSW Australia; 2 Graduate School of Health, University of Technology Sydney, Sydney, NSW Australia

**Keywords:** Eating disorders, Path analysis, Theoretical model, Binge eating, Compensatory behaviours

## Abstract

**Purpose:**

This study aimed to validate a core beliefs model of disordered eating in a sample of adults with a current eating disorder diagnosis. This model outlines important processes and pathways from maladaptive eating disorder core beliefs to dietary restraint, objective binge eating and compensatory behaviours.

**Methods:**

Participants were adults (*N* = 232) living in English-speaking countries who self-reported having a current eating disorder diagnosis given by a healthcare professional. Preliminary analyses included examining correlations between included variables and internal consistency. Path analysis was conducted in R to test the core beliefs model.

**Results:**

The original model demonstrated poor to acceptable fit to the observed data. Minor modifications were utilised to remove non-significant paths to improve fit, including the removal of ‘perfectionism’ as a mediating variable in the model. The final modified model indicated acceptable model fit. This model demonstrates specific pathways that maladaptive core beliefs contribute to the development of dietary restraint, objective binge eating and compensatory behaviours, through either increased pre-occupation with eating, weight and shape, or through increased negative affect, emotional dysregulation and meta-cognitive beliefs about binge eating.

**Conclusions:**

The present study provides partial validation of a core beliefs model of disordered eating and extends the current understanding of how maladaptive core beliefs may impact the development of key disordered eating symptomatology.

Level of Evidence: IV.

Core beliefs are rigid, unconditional beliefs about the self, others, or the world, that often present as maladaptive and inflexible, and are thought to arise from difficult early life experiences [1]. For individuals with eating disorders (EDs), maladaptive core beliefs often present as negative, dysfunctional beliefs about the self [1]. These beliefs have been identified as critical factors in the development and maintenance of disordered eating symptomatology [1, 2]. However, despite this, evidence-based treatments for EDs do not consistently prioritise the assessment and treatment of these beliefs [3]. The increasing prevalence of disordered eating symptomatology, high comorbidity in EDs, and ongoing low ED remission rates indicates that more attention to maladaptive core beliefs during assessment and treatment is warranted [4, 5].

The transdiagnostic maintenance model of EDs [6], the cognitive model of bulimia nervosa [2], and the schema-focused cognitive behavioural model of EDs [1], each include a 'core belief' type theoretical component. However, though suggested by seminal researchers in this area [1, 2], until recently, no single theoretical model or evidence-based treatment has provided a clear, comprehensive and wholistic understanding of how maladaptive core beliefs contribute to the development and maintenance of EDs, nor whether specific dimensions of core beliefs differentially impact these processes [5]. The core beliefs model of disordered eating was developed in response to the lack of a cohesive model to delineate the specific pathways and processes from ED core beliefs to key disordered eating symptoms [7]. This model describes the various pathways from a general ED core belief construct to three core clusters of ED behaviours; dietary restraint, objective binge eating (OBE), and compensatory behaviours (see Fig. 1). The model outlines two possible pathways from negative ED core beliefs to dietary restraint, either through increased perfectionistic attitudes, which in turn lead to increased pre-occupation with eating, weight and shape, ultimately resulting in increased dietary restraint, or instead directly through this pre-occupation, without perfectionism as a necessary mediating variable. [7]

**Fig. 1:**
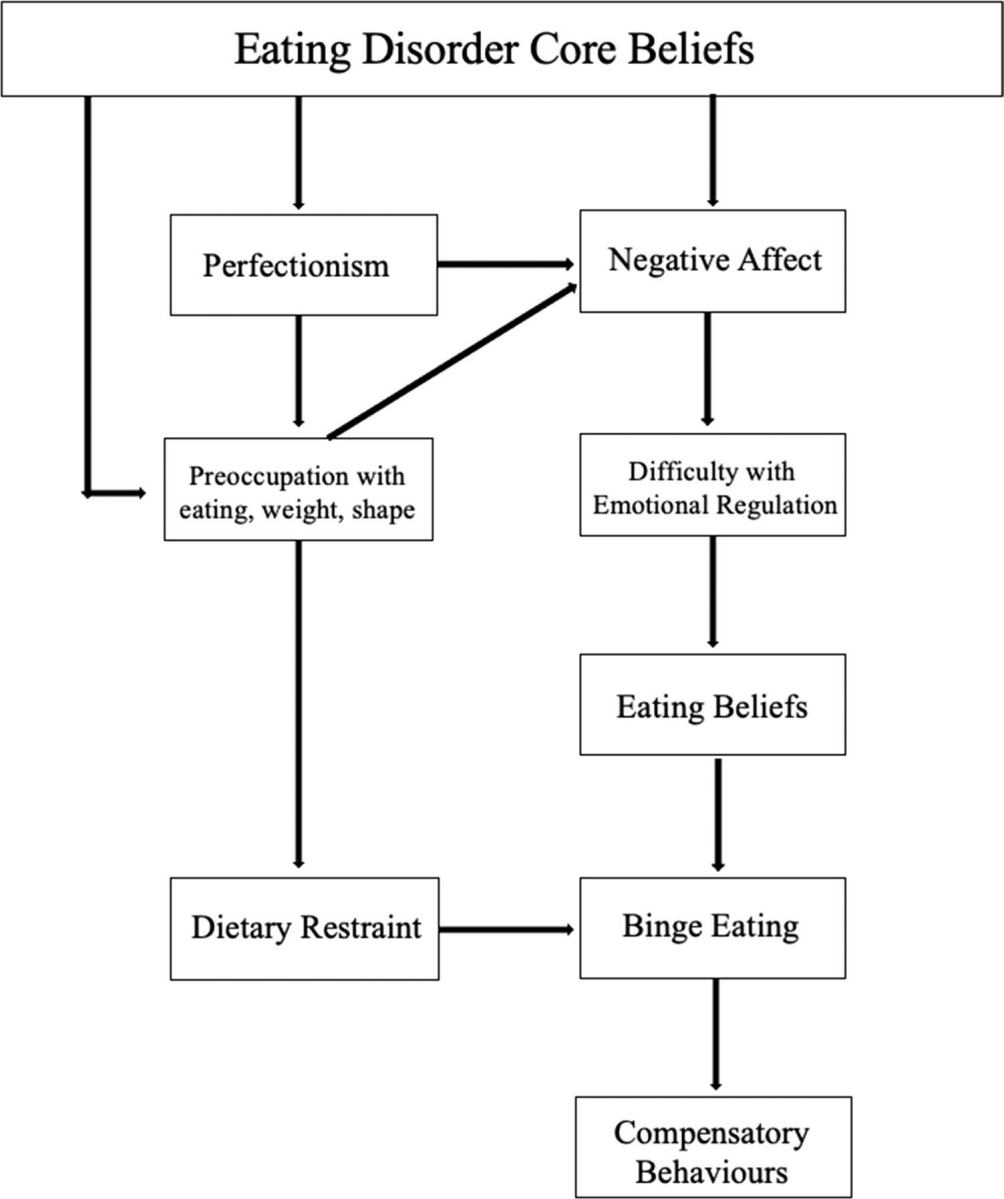
Core beliefs model of disordered eating

Moreover, the model describes possible pathways to OBE and compensatory behaviours through either of the two major pathways (i.e., resulting from increased dietary restraint), or via an emotion regulation pathway. First, the activation of ED core beliefs (e.g., ‘I am repulsive’) may contribute to or trigger increased negative affect (e.g., depression, anxiety, or stress) and subsequent difficulties with emotional regulation, which in turn may result in increased maladaptive meta-cognitive beliefs about binge eating, to help regulate these emotions [7]. Activation of meta-cognitive beliefs about eating (e.g., ‘Eating means I don’t have to think about negative things’) can result in OBE, and possible consequent compensatory behaviours. However, this particular process may also occur after an increase in perfectionistic attitudes and/or over-evaluation of eating, weight and shape that occur as a result of increased maladaptive ED core beliefs [7]. Therefore, the model delineates several paths of possible mediating variables and processes that subsequently result in any of three central disordered eating behaviours.

Overall, this model integrates key constructs from existing models that include a 'core belief' type component, to provide a more comprehensive and nuanced explanation of the processes that may contribute to increased disordered eating symptoms. However, the core beliefs model was initially developed in a non-clinical sample, including those with sub-clinical or prodromal disordered eating. To determine its applicability and generalisability to clinical populations, it is important to validate the model in individuals with clinical levels of disordered eating. As such, the current study aimed to validate the core beliefs model of disordered eating in a sample of individuals who reported having a current ED diagnosis. We predicted the core beliefs model would show acceptable to good fit to the data, and thus would demonstrate evidence for model validity in the current sample. Validation of this model would support ongoing rationale for emphasising the assessment of maladaptive ED core beliefs during intervention, and the importance of early identification of these beliefs, which can be monitored as treatment targets. Figure 1 illustrates the core beliefs model.

## Methods

### Participants

Participants were an international sample of adults from English-speaking countries who self-reported having a current ED diagnosis (e.g., anorexia nervosa [AN], other specified feeding or eating disorder [OSFED]). Participants were invited to the study if they lived in an English-speaking country, were 17 years of age or older, and reported having a current ED diagnosis from a healthcare professional. From an initial pool of 590 participants, 433 provided complete responses (i.e., not missing full measures). Of these, 194 were excluded from analyses (*n* = 28 failed the quality assurance item; *n* = 122 did not report having a current ED diagnosis; *n* = 45 did not reside in an English-speaking country, *n* = 6 were younger than 17 years). The final sample for analyses consisted of 232 participants (79.3% female; M age = 29.04 years, SD = 9.36 years; Country: Australia [17.7%], Canada [3.0%], Republic of Ireland [5.6%], New Zealand [1.7%], United Kingdom [16.4%], United States [55.6%]). The sample comprised of 93 individuals who reported a current diagnosis of AN restricting subtype (AN-R; 40.1%), 18 with AN binge–purge subtype (AN-BP; 7.8%), 26 with bulimia nervosa (BN; 11.2%), 33 with binge eating disorder (BED; 14.2%), 19 with OSFED–AN (8.2%), 19 with OSFED–BN (8.2%), 14 with OSFED–BED (6%), and 10 with avoidant/restrictive food intake disorder (ARFID; 4.3%). To conduct path analysis with sufficient statistical power, we aimed to obtain the recommended minimum sample size (*N* = 200 to 500), based off estimates from simulation studies and guidelines for conducting structural equation modelling (SEM) with ordinal data [8]. The final sample included in statistical analyses ensured sufficient power was obtained.

## Measures

### Eating disorder core beliefs questionnaire revised (ED-CBQ-R)

The 15-item ED-CBQ-R [9] assessed the overall construct of ‘ED Core Beliefs’. Items were rated on a seven-point scale (Feels very much untrue [1] to Feels very much true [7], e.g., ‘Inhibited’, ‘Abandoned’, ‘Putrid’, ‘Needy’). Higher scores indicate higher maladaptive core beliefs (range 1–7). The scale has previously demonstrated acceptable reliability and validity [9], and demonstrated good internal consistency in the current sample (Ω = 0.81).

### Eating disorder examination questionnaire (EDE-Q)

The 28-item EDE-Q [10] was used to assess ‘Preoccupation with eating, weight and shape’ (eating concerns, weight concerns and shape concerns subscales), and ‘Dietary restraint’ (dietary restraint subscale), where items were rated on a seven-point scale (No days [0] to Every day [6], range 0–6). It was also used to assess ‘Objective Binge eating’, and ‘Compensatory Behaviours’. Item 13 was used to assess ‘Objective Binge Eating’ and Item 16 used to assess ‘Compensatory behaviours’, to assess frequencies over the last 28 days.^1^ In the current sample, the preoccupation score (Ω = 0.91) and dietary restraint subscale (Ω = 0.83) had good internal consistency.

### Eating beliefs questionnaire 18 (EBQ-18)

The 18-item EBQ-18 [11] assessed ‘Eating Beliefs’ including positive, negative and permissive meta-cognitive beliefs about binge eating (e.g., ‘There is nothing I can do to stop eating’). Items were rated on a scale from strongly disagree (1) to strongly agree (5), where higher scores indicate higher meta-cognitive beliefs (range 1–5). The EBQ-18 has previous demonstrated good psychometric properties, including validity, reliability, and clinical utility [12], and demonstrated good internal consistency in the current sample (Ω = 0.95).

### Frost multidimensional perfectionism scale—brief (FMPS-B)

The eight-item FMPS-B [13] assessed the construct of ‘Perfectionism’. Items are rated on a five-point scale (e.g., ‘The fewer mistakes I make, the more people will like me’). Higher scores indicate higher perfectionism (Strongly disagree [1] to strongly agree [5], range 1–5). This scale has been validated in both community and clinical samples [13], and displayed good internal consistency in the current sample (Ω = 0.82).

### Depression anxiety stress scales (DASS-21)

The DASS-21 [14] was used to assess ‘Negative Affect’. Items were rated on a four-point scale (e.g., ‘I found it difficult to work up the initiative to do things’). Higher scores indicated higher negative affect (Did not apply to me at all [0] to Applied to me very much/most of the time [3], range 0–3). This measure has been cross-culturally validated [15], and displayed good internal consistency in this sample (Ω = 0.87).

### Difficulties in emotion regulation scale brief version (DERS-16)

The 16-item DERS-16 [16] assessed ‘Difficulties with Emotional Regulation’. Items were rated on a five-point scale (e.g., ‘When I am upset, I become out of control’). Higher scores reflect greater difficulties with emotion regulation (range 1–5; 1 [Almost never] to 5 [Almost always]). The DERS-16 has previously demonstrated good reliability and validity [16], and displayed good internal consistency in the current sample (Ω = 0.92).

## Procedure

Ethics approval was obtained from The University of Sydney Human Research Ethics Committee (Project Code: 2022/856), and all procedures were conducted in accordance with these ethical standards. Participants were recruited through a range of state-based, national, and international ED associated institutions (e.g., National Eating Disorders Collaboration [Australia], BodyWhys [Republic of Ireland], Inside Out Institute [Australia]) and social media sites (e.g., Instagram and Reddit). Participation was incentivised by entering a prize draw to win one of the four $50 gift vouchers. Participants provided informed consent before voluntarily completing the online test battery using Qualtrics. Demographic information was collected for participants including age, gender, current country of residence, and ED diagnostic status as indicated by a mental healthcare professional. The study was not pre-registered, though its procedures follow that of the model development paper. [7]

### Statistical analyses

Statistical analyses were conducted using R Project for Statistical Computing and IBM Statistical Package for Social Sciences (SPSS) Statistics (version 26.0). Preliminary analyses were conducted before testing the model, including examining Kendall’s Tau (τ) correlations to examine the relationships between included variables, and internal consistency of scales using McDonald’s Omega (Ω) (values > 0.70 and < 0.95 were considered acceptable) [17]. Mean imputation was utilised to handle missing data for participants (*n* = 8) with responses with less than 10% missing data per scale (no cases missing > 10%). [18]

To test the core beliefs model, path analysis was conducted in R using weighted least squares with a mean and variance adjusted test statistic (WLSMV) as the robust estimation method [19]. Similar to the statistical analysis plan outlined in detail in Hatoum et al. [7], the general model was tested using the following values to evaluate model fit; comparative fit index (CFI) or Tucker–Lewis index (TLI) of ⩾ 0.95 was considered good and ⩾ 0.90 acceptable, and an RMSEA value of ⩽ 0.050 considered good and ⩽ 0.080 acceptable. [20]

## Results

### Descriptive statistics and correlations

In terms of the correlations expected and predicted in the model, as represented by each of the hypothesised paths, most variables displayed the expected positive correlations (Table 1). However, ‘ED Core Beliefs’ did not correlate with ‘Perfectionism’ as anticipated, nor was there an expected correlation between ‘Dietary Restraint’ and ‘OBE’, see Table 1 for all descriptive statistics (mean and SDs) for included variables and correlations between variables.

**Table 1:** Descriptive statistics and Kendall’s Tau correlations for all included variables

	Mean (SD)	1	2	3	4	5	6	7	8
1. Eating Disorder Core Beliefs	4.50 (1.00)								
2. Perfectionism	3.84 (0.78)	0.09							
3. Preoccupation	4.21 (1.15)	0.21**	0.19**						
4. Negative Affect	1.78 (0.52)	0.29**	0.11*	0.26**					
5. Difficulty with Emotional Regulation	3.56 (0.86)	0.35**	0.17**	0.23**	0.51**				
6. Eating Beliefs	2.49 (1.04)	0.16**	− 0.15**	0.08	0.05	0.15**			
7. Dietary Restraint	3.86 (1.63)	0.10*	0.18**	0.44**	0.11*	0.04	− 0.11*		
8. Objective Binge Eating	8.37 (13.76)	0.14**	− 0.14**	0.16**	0.04	0.13**	0.57**	-0.03	
9. Compensatory Behaviours	7.20 (18.67)	0.13*	− 0.03	0.18**	0.10*	0.13*	0.31**	0.11*	0.43**

Preoccupation refers to preoccupation with eating, weight, and shape. SD = standard deviation

^**^p < .01 (two-tailed), *p < .05 (two-tailed)

### Path analysis

**Fig. 2:**
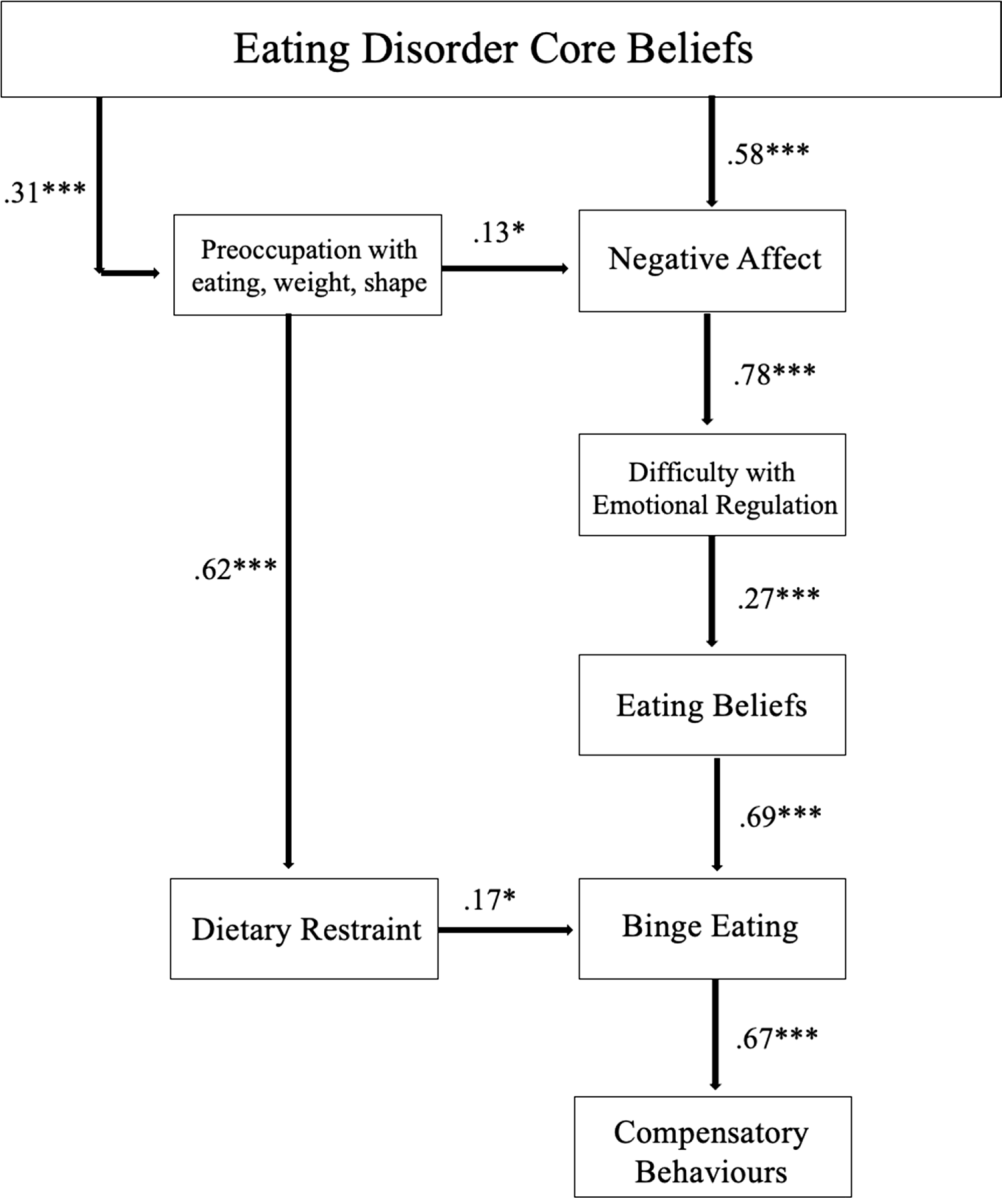
Core beliefs model of disordered eating. Regression coefficients were standardised. * p < .05. ** p < .01. *** p < .001

Path analysis was conducted in R using WLSMV estimation to test if the proposed model fit the data. The original model (Fig. 1) demonstrated poor to acceptable fit to the observed data (χ^2^ [df] = 83.10 [24], CFI = 0.91, TLI = 0.86, RMSEA [90% CI] = 0.11 [0.09, 0.13]). Minor modifications were utilised to remove non-significant paths to improve fit. The path from ‘ED Core Beliefs’ to ‘Perfectionism’ was not significant, necessitating its exclusion in the model, and the removal of subsequent paths from ‘Perfectionism’ to other variables in the model (see Fig. 2). Following this modification, the final modified model had acceptable fit for all fit statistics, apart from RMSEA (χ^2^ [df] = 54.84 [19], CFI = 0.94, TLI = 0.91, RMSEA [90% CI] = 0.10 [0.08, 0.13]).

## Discussion

The present study aimed to validate the core beliefs model of disordered eating [7] in a sample of adults from English-speaking countries who reported having a current diagnosis of an ED. We found partial support for the original model. The hypothesised path between ‘ED core beliefs’ and ‘Perfectionism’ was not significant, and as such ‘Perfectionism’ was removed as a mediating variable. This did not impact the remaining serially mediating variables or the model, as there was a direct relationship between ED core beliefs and both ‘Preoccupation with eating, weight, shape’ and ‘Negative Affect’. Previous theoretical models [1, 2, 6] and the previous test of the original model [7] suggest that ED core beliefs can certainly contribute to the development of ED symptomatology even in the absence of perfectionism. Nevertheless, the lack of relationship between perfectionism and maladaptive core beliefs in the present study was unexpected and contrary to previous theory and study [6]. It is possible that distinct dimensions of perfectionism have differing relationships with ED symptom profiles. For example, meta-analytic data have outlined that only perfectionistic concerns, but not perfectionistic strivings, display a strong relationship with binge eating [21]. As almost half of the present sample (47.3%) reported a diagnosis with binge eating as a core feature, it is possible that the mixed sample affected the measurement of perfectionism in the present study. In future, testing the model in specific diagnostic groups could help assess how different dimensions of perfectionism may be differentially related to variables in the model.

The final model displayed acceptable fit to the data. The path from ‘ED core beliefs’ was significant for both ‘Preoccupation with eating, weight, shape’ and ‘Negative Affect’. The pathways from ‘Preoccupation with eating, weight, shape’ was significant to both ‘Dietary Restraint’ and ‘Negative Affect’. Moreover, the path from ‘Dietary Restraint’ was significant to ‘Binge Eating’, which in turn was to ‘Compensatory Behaviours’. The model also displays the alternative path to the development of binge eating and compensatory behaviours through a series of mediating variables from ‘Negative Affect’ to ‘Eating Beliefs’. As in the development paper [7], the model in the present study highlights how maladaptive core beliefs may contribute to the development of dietary restraint, objective binge eating and compensatory behaviours, through either (or potentially both) increased pre-occupation with eating, weight and shape, or through increased negative affect, emotional dysregulation and meta-cognitive beliefs about binge eating. Altogether, the model outlines the many pathways that may be activated and implicated in the development of ED symptomatology for any given individual.

We provided partial validation of a core beliefs model of disordered eating in a sample of adults with a current ED diagnosis. The outcomes of the present study help enhance our understanding of the complex pathways contributing to ED symptomatology and provide a more nuanced framework for clinicians and researchers to assess and understand the development and maintenance of disordered eating behaviours. This framework aims to inform more comprehensive case formulation by promoting routine and early identification of core beliefs in individuals presenting for treatment, as well as evaluating the influence of these beliefs on other maintenance mechanisms within individual formulations. It is hoped this may enhance precision in treatment planning.

However, some limitations must be acknowledged and addressed in future study. Primarily, the study relied on self-reported ED diagnoses, as data were not collected from treatment sites. Future study should seek to validate the model in a treatment-seeking sample. Furthermore, the present study collected cross-sectional data. Utilising ecological momentary assessment could allow for longitudinal examination of these processes at the state level, potentially offering a more nuanced understanding of the maintenance mechanisms not yet captured in the current model. Future work could assess non-recursive model to explore bi-directional relationships between variables. It would be also valuable to evaluate the core beliefs model in specific diagnostic subgroups (e.g., AN-R vs. BED) to determine its generalizability and specificity. Addressing these areas will be crucial for refining and validating the proposed model. Finally, the present study attempted to reduce sample heterogeneity by only seeking to include participants from English-speaking countries. This limits generalisability of the model cross-culturally. Future research could assess whether the processes and pathways to ED symptomatology from maladaptive core beliefs are comparable across cultures. Altogether, the present study provides partial support for the core beliefs model of disordered eating, which highlights several nuanced processes and pathways from ED core beliefs to dietary restraint, binge eating and compensatory behaviours.

## Strength and limits

A key strength of this study is the use of path analysis with well-validated measures to empirically evaluate a comprehensive theoretical model of core beliefs in a relatively large, international sample of adults with self-reported eating disorders. However, the cross-sectional design precludes causal inferences, and reliance on self-reported diagnoses may limit diagnostic accuracy. In addition, the exclusion of those not residing in English-speaking countries restricts the generalisability of the findings across cultures.

### What is already known on this subject?

The core beliefs model of disordered eating attempted to create cohesion between key pathways in models that include a ‘core belief’ type component, but with greater theoretical depth by presenting several nuanced processes that may result in increased disordered eating symptomatology. The model describes possible pathways to objective binge eating and compensatory behaviours through either of the two major pathways, increased dietary restraint, or via an emotion regulation pathway.

### What this study adds?

This study provides partial empirical validation for a core beliefs model of disordered eating, highlighting multiple pathways from maladaptive core beliefs to key eating disorder behaviours via preoccupation with eating, weight and shape, and negative affect. These findings reinforce importance of assessing and targeting maladaptive core beliefs in both early intervention and treatment of eating disorders.

## Data Availability

The data using during the current study is available from the corresponding author on reasonable request.

## Footnotes

1 During structural equation modelling, items 13 and 16 were standardised to create scores on a 7-point scale: 0 = 0, 1 = 1 – 3, 2 = 4 – 10, 3 = 11 – 20, 4 = 21 – 28, 5 = 29 – 5, 6 = 50 + .

## References

[1] Waller GKH, Ohanian V (2007) Schema-focused cognitive-behavioral therapy for eating disorders. In: Riso LP, du Toit PL, Stein DJ, Young JE (eds) Cognitive schemas and core beliefs in psychological problems: a scientist-practitioner guide. American Psychological Association, Washington (DC), pp 139–175

[2] Cooper M, Wells A, Todd G (2004) Cognitive model of bulimia nervosa. Br J Clin Psychol Br Psychol Soc 43:1–16 10.1348/014466504772812931 15005903

[3] Dalle Grave R, Pike KM (2023) Cognitive Behavioral Therapy and Eating Disorders. In: Robinson P, Wade T, Herpertz-Dahlmann B, Fernandez-Aranda F, Treasure J, Wonderlich S (eds) Eating Disorders: An International Comprehensive View. Springer International Publishing, Cham, pp 1–15

[4] Levinson CA, Cusack C, Brown ML, Smith AR (2022) A network approach can improve eating disorder conceptualization and treatment. Nat Rev Psychol 1(7):419–430 36330080 10.1038/s44159-022-00062-y PMC9624475

[5] Pennesi JL, Wade TD (2016) A systematic review of the existing models of disordered eating: do they inform the development of effective interventions? Clin Psychol Rev 43:175–192 26781985 10.1016/j.cpr.2015.12.004

[6] Fairburn CG, Cooper Z, Shafran R (2003) Cognitive behaviour therapy for eating disorders: a “transdiagnostic” theory and treatment. Behav Res Ther 41(5):509–528 12711261 10.1016/s0005-7967(02)00088-8

[7] Hatoum AH, Burton AL, Abbott MJ (2024) A core beliefs model of disordered eating: processes and pathways to eating disorder symptomatology. J Eat Disord 12(1):203 39696627 10.1186/s40337-024-01167-w PMC11657716

[8] Thakkar J. Procedural Steps in Structural Equation Modelling. 2020. 29–34.

[9] Hatoum AH, Burton AL, Abbott MJ (2022) Assessing negative core beliefs in eating disorders: revision of the eating disorder core beliefs questionnaire. J Eat Disord 10(1):18 35144689 10.1186/s40337-022-00542-9 PMC8830168

[10] Fairburn CG, Beglin SJ (1994) Assessment of eating disorders: interview or self-report questionnaire? Int J Eat Disord 16(4):363–370 7866415

[11] Burton AL, Abbott MJ (2018) The revised short-form of the eating beliefs questionnaire: measuring positive, negative, and permissive beliefs about binge eating. J Eat Disord 6(1):37 30450206 10.1186/s40337-018-0224-0 PMC6219185

[12] Burton AL, Mitchison D, Hay P, Donnelly B, Thornton C, Russell J et al (2018) Beliefs about binge eating: psychometric properties of the eating beliefs questionnaire (EBQ-18) in eating disorder, obese, and community samples. Nutrients. 10.3390/nu10091306 30223500 10.3390/nu10091306 PMC6165353

[13] Burgess AM, Frost RO, DiBartolo PM (2016) Development and validation of the Frost Multidimensional Perfectionism Scale-Brief. J Psychoeduc Assess 34(7):620–633

[14] Lovibond PF, Lovibond SH (1995) The structure of negative emotional states: comparison of the Depression Anxiety Stress Scales (DASS) with the Beck Depression and Anxiety Inventories. Behav Res Ther 33(3):335–343 7726811 10.1016/0005-7967(94)00075-u

[15] Zanon C, Brenner RE, Baptista MN, Vogel DL, Rubin M, Al-Darmaki FR et al (2021) Examining the dimensionality, reliability, and invariance of the Depression, Anxiety, and Stress Scale-21 (DASS-21) across eight countries. Assessment 28(6):1531–1544 31916468 10.1177/1073191119887449

[16] Bjureberg J, Ljótsson B, Tull MT, Hedman E, Sahlin H, Lundh LG et al (2016) Development and validation of a brief version of the difficulties in emotion regulation scale: the DERS-16. J Psychopathol Behav Assess 38(2):284–296 27239096 10.1007/s10862-015-9514-x PMC4882111

[17] Dunn TJ, Baguley T, Brunsden V (2014) From alpha to omega: a practical solution to the pervasive problem of internal consistency estimation. Br J Psychol 105(3):399–412 24844115 10.1111/bjop.12046

[18] Lee JH, Huber JC Jr (2021) Evaluation of multiple imputation with large proportions of missing data: how much is too much? Iran J Public Health 50(7):1372–1380 34568175 10.18502/ijph.v50i7.6626 PMC8426774

[19] Sellbom M, Tellegen A (2019) Factor analysis in psychological assessment research: common pitfalls and recommendations. Psychol Assess 31(12):1428–1441 31120298 10.1037/pas0000623

[20] Schreiber JB, Nora A, Stage FK, Barlow EA, King J (2006) Reporting structural equation modeling and confirmatory factor analysis results: a review. J Educ Res 99(6):323–338

[21] Vicent M, Gonzálvez C, Quiles MJ, Sánchez-Meca J (2023) Perfectionism and binge eating association: a systematic review and meta-analysis. J Eat Disord 11(1):101 37365626 10.1186/s40337-023-00817-9 PMC10294348

